# Synopsis of the pharmacokinetics, pharmacodynamics, applications, and safety of firocoxib in horses

**DOI:** 10.1016/j.vas.2023.100286

**Published:** 2023-01-11

**Authors:** Charbel Fadel, Mario Giorgi

**Affiliations:** a Department of Veterinary Medicine, University of Sassari, Sassari, Italy; b Department of Veterinary Sciences, University of Pisa, Pisa, Italy

**Keywords:** Coxib, Firocoxib, Horses, Pain management, Pharmacodynamics, Pharmacokinetics

## Abstract

According to *in vitro* and *in vivo* investigations, firocoxib (FX), a second-generation coxib, is a highly selective COX-2 inhibitor in horses. With a COX-1/COX-2 IC_50_ ratio of 643 in horses, FX spares the COX-1 inhibitory effects. It is approved for the treatment of musculoskeletal problems and lameness in horses and dogs with osteoarthritis (OA). For the treatment of OA in horses, both an injectable formulation for IV administration at a dose of 0.09 mg/kg for five days and an oral paste formulation at a dose of 0.1 mg/kg for 14 days are licensed. Numerous analytical methods were reported in the literature to quantify FX in biological fluids, using HPLC and LC-MS. FX presents remarkable pharmacokinetics and pharmacodynamics compared to other coxibs. It has an oral bioavailability of 80% or higher and is effectively absorbed by horses. Its volume of distribution is around 2 L/kg, and it is slowly eliminated. Due to the long elimination half-life (around 2 days), which allows a once daily dosing, a single 0.3 mg/kg loading dose has been recommended. This enables the establishment of steady-state drug concentrations within 24 h, making it appropriate for acute treatment as well. Its IC_80_ is equal to 103 ng/mL in whole blood and, with an EC_50_ of 27 ng/mL, it has the highest affinity for its receptor compared to the other commonly administered NSAIDs in horses.

## Introduction

For medical, ethical, and welfare reasons, the veterinary surgeon's responsibility includes assessing and alleviating pain in animals. Pain assessment in large animals has received less attention than in small animals. This is the case in horses as well, where pain management has gotten little attention until lately (Taylor, 2002). There may be two main reasons for this; i) difficulty in assessing and quantifying pain in these species and ii) lack of data regarding the efficacy and dose for analgesics. Recently, unresolved stress or pain behavior were identified as one of the four priority welfare challenges facing horses in the United Kingdom (Horseman, 2017).

Pain is difficult to define in animals since it is a subjective emotional experience as well as a sensory (objective) one. As is the case for small animals, there is no single parameter available to objectively assess pain in horses (Taylor et al., 2002). Parameters used to assess pain have included cardiovascular measurements such as heart rate and blood pressure, and plasma concentrations of β-endorphins, catecholamines, and corticosteroids (Valverde & Gunkel, 2005). Other means include elaborated methods such as force plate and gait analysis. Furthermore, some studies often measure nociceptive responses using electrical, mechanical or thermal stimuli (Moens et al., 2003).

Inflammation is a beneficial response that protects tissues affected by injury or pathogens; yet, left uncontrolled, inflammation (and inflammatory mediators) can contribute to the pathogenesis of numerous diseases including arthritis, laminitis, cancer, periodontitis-induced bone loss, and colitis (Steele, 2003). This is why administration of analgesics or pain killers is required on welfare grounds, especially since that prolonged stimulation of the pain pathways can result in serious alterations often referred to as peripheral and central sensitization (‘wind-up’) (Love, 2009).

Along the major groups of analgesics, non-steroidal anti-inflammatory drugs (NSAIDs) are used in human and veterinary medicine to control inflammation and pain. Traditional or non-selective NSAIDs inhibit both COX isoforms, COX-1 and COX-2, and are efficacious in reducing pain and inflammation, but then are also associated with irritation of gastric mucosa. The use of these NSAIDs has other side effects as well, such as on renal function, including renal papillary necrosis, interstitial nephritis, fluid retention, and electrolyte imbalances such as hyperkalemia (Whelton & Hamilton, 1991).

Hence, the several complications of the non-selective NSAIDs, alongside the recognition that inhibition of COX-2 might be sufficient to achieve the anti-inflammatory benefits of NSAIDs, the growing interest in animal welfare, and the higher demanded level of care by the animals’ owners, steered towards the development of the coxibs, that would selectively inhibit only COX-2, and would have a better safety profile (Kim & Giorgi, 2013).

Being launched first in human medicine, it followed that many selective inhibitors were introduced into clinical use for the veterinary market, such as deracoxib (2002), firocoxib (FX) (2007), mavacoxib (2008) and robenacoxib (2009) (Bergh & Budsberg, 2005). Cimicoxib (2011) has also been introduced for the veterinary market from the human field (Emmerich, 2012). Lastly, enflicoxib (2021) is the most recently licensed coxib, in dogs (Pye et al., 2022).

FX (3-(cyclopropylmethoxy)−4-(4-methylsulfonylphenyl)−5,5-dimethylfuranone) is a second-generation coxib and a highly selective COX-2 inhibitor in horses. It is labelled for the management of musculoskeletal pain and lameness associated with osteoarthritis (OA) in horses and dogs (McCann et al., 2004).

In horses, FX has been demonstrated to alleviate osteoarthritic pain, to not impair jejunal mucosal regeneration after ischemia-induced injury, and to permeate the aqueous humor and synovial fluid (Cook et al., 2009; Doucet et al., 2008; Hilton et al., 2011; Koene et al., 2010; Kvaternick et al., 2007; Orsini et al., 2012). It is licensed for use in horses as an oral paste formulation and an intravenously injected solution for the relief of pain and inflammation caused by equine OA or degenerative joint disease in horses over the age of one year (Equioxx® Package Insert, Merial, The Animal Health Division of Sanofi, Duluth, GA, USA).

This review provides an overview of the current knowledge on FX pharmacology in horses, with a focus on its detection methods, pharmacokinetics (PK), pharmacodynamics (PD), safety profile, medical applications, and potential interaction with other drugs.

## Methodology and literature search

Electronic databases of published scientific literature were the main source for this review. PubMed, Scopus, and Google Scholar were searched for *in vitro* and *in vivo* research findings, as well as pharmacokinetic and pharmacodynamic studies, and safety evaluation studies. Additional articles of interest were obtained through cross-referencing of published literature. The primary key terms used were “firocoxib in horses” “pharmacokinetics”, “pharmacodynamics,” “drug interactions”, “safety evaluation” and “analytical methods”. Only English language papers were taken into consideration.

All of the firocoxib PK studies found in horses were included in this study, with no exclusions, as they all met the prerequisites; no inconsistencies were discovered between the studies; plasma concentrations were measured in accordance to the international guidelines; no duplicate results were found; and the results were comprehensible in all of the selected studies, as they were consistent with one another and had comparable PK parameter values. They were all applied on healthy horses, with some variables in the animals used (breed, feeding status, age, sex) mentioned in Table 3.

## Description and physicochemical properties

**Fig. 1:**
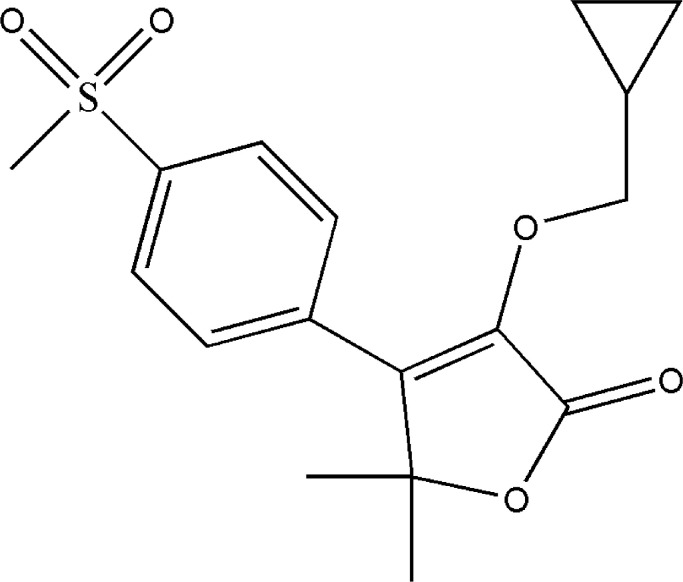
Chemical structure of firocoxib.

The IUPAC name is 3-(cyclopropylmethoxy)−5,5-dimethyl-4-(4-methylsulfonylphenyl)furan-2-one. FX is practically insoluble in water (0.0105 mg/mL), soluble in ethanol (3 mg/mL), and highly soluble in dimethyl sulfoxide (67 mg/mL). The chemical properties are listed in Table 1, and the chemical structure is depicted in Fig. 1.

**Table 1:** Chemical features of firocoxib.

Substance origin	Synthetic
Molecular target	[Prostaglandin G/H Synthase 2, antagonist]
Boiling Point	563.9 °C at 760 mmHg
Synonyms	Equioxx; Equixx; Librens; ML 1,785,713; Previcox; Firocoxib– GMP; TIANFU CHEM—Firocoxib; CS-675
Density	1.31 g/cm^3^
IUPAC Name	3-(cyclopropylmethoxy)−5,5-dimethyl-4-(4-methylsulfonylphenyl)furan-2-one.
Melting Point	78–80 °C
Molar Mass	336.4 g/mol
Molecular Formula	C_17_H_20_O_5_S
Dissociation constant	pka= 19.69 (Strongest Acidic)
Storage	3 years −20 °C in powder form; 2 years −80 °C in solvent

## Previous researches and described analytical methods

Although only approved for use in horses and dogs, several studies have assessed FX's pharmacological patterns in other animal species such as camels, sows, goats, rabbits, alpacas, and calves (Chadwick, 2017; Wasfi et al., 2015; Stuart et al., 2019; Gardhouse et al., 2022; Kleinhenz et al., 2020; Stock et al., 2014). In prior researches, the analytical techniques for detecting FX concentrations were held with High Performance Liquid Chromatography (HPLC) and Liquid Chromatography Mass Spectrometry (LC-MS). In fact, the HPLC was extensively used for estimation of COX-2 inhibitors in biological fluids (for most of the published methods) (Rao et al., 2005). The majority of the researchers have used the reversed-phase mode with ultraviolet (UV) absorbance detection (at 290 nm) because it provided the best available reliability, repeatability, analysis time and sensitivity. Other detectors, such as fluorescence, with an excitation wavelength of 250 nm and an emission wavelength of 375 nm, were also used to assess and control the COX-2 inhibitors purity. Furthermore, LC-MS was also used to detect not only the majority of COX-2 inhibitors metabolites in urine and plasma, but also the degradation products of bulk drugs and formulations. The same procedures apply categorically to FX.

FX is a small non-ionizable molecule, and has a conjugated ring system similar to other coxibs making it amenable to UV and fluorescence detection (Kvaternick et al., 2007). While the LC-MS methods generally reported much lower limits of quantitation for the coxibs, ranging from <1–10 ng/mL, sufficient limits of quantitation (5–25 ng/mL) were reported for HPLC-UV methods of related compounds (Ramakrishna et al., 2006).

For sample preparation of FX, the published methods employed protein precipitation, liquid–liquid extraction and solid-phase extraction, as seen in Table 2. Typically solvents such as ethyl acetate/hexane or acetonitrile were reported for liquid–liquid extraction or protein precipitation, respectively. While others reported the use of solid-phase extraction, mainly reversed-phase, for the separation of FX from plasma. Finally regarding the internal standard, deracoxib was generally utilized in the earlier studies.

**Table 2:** Summary of the analytical methods used for firocoxib quantification reported in the literature.

Reference	Biologi**ca**l matrix	LC + Detector	Column	Mobile phase	Clean-up method	LOD	LLOQ
Cox and Yarbrough, 2011	Tissues	HPLC-UV	C18 (4.6 mm x 150 mm, 3.5 µm)	H2O with 0.025% trifluoroacetic acid and ACN (50:50, v/v)	Liquid-liquid extraction, ethyl acetate:hexane (40:60, v/v)	NA	5 ng/g
Cox et al., 2012	Plasma	HPLC-UV	C18 (4.6 mm x 150 mm, 5 µm)	H2O with 0.025% trifluoroacetic acid and ACN (50:50, v/v)	Liquid-liquid extraction, ethyl acetate:hexane (40:60, v/v)	NA	5 ng/mL
Cox et al., 2013	Plasma	HPLC-Fluorescence	C18 (4.6 mm x 150 mm, 5 µm)	methanol:water (51:49 v/v)	Protein precipitation, ACN	NA	2.5 ng/mL
Wilson et al., 2017	Plasma	HPLC-Fluorescence	C18 (4.6 mm x 250 mm, 5 µm)	methanol:water (51:49 v/v)	Protein precipitation, ACN/water (40:60, v/v)	NA	2.5 ng/mL
Letendre et al., 2008	Plasma (after IV administration)	HPLC-UV	ODS-3 (4.6 mm x 150 mm, 5 µm)	ACN/water/trifluoroacetic anhydride (45:55:0.025 v/v/v)	Solid-phase extraction with water and ACN	10 ng/mL	25 ng/mL
	Plasma + urine (after oral administration)	LC-MS/MS	Phenyl-Hexyl (2 mm × 100 mm, 3 µm)	ACN:aqueous buffer (2 mM ammonium formate + 0.1% formic acid)(45:55, v/v)	Solid-phase extraction with water and MeOH	0.25 ng/mL	10 ng/mL for plasma; 5 ng/mL for urine
Kvaternick et al., 2007	Plasma	HPLC-UV	ODS-3 (4.6 mm x 150 mm, 5 µm)	ACN and 0.25% trifluoroacetate anhydride in H2O (45:55, v/v)	Solid-phase extraction with water and ACN	10 ng/mL	25 ng/mL
Letendre et al., 2007	Plasma + urine	LC-MS/MS	Phenyl-Hexyl (2 mm × 100 mm, 3 µm)	ACN:aqueous buffer (2 mM ammonium formate + 0.1% formic acid)(45:55, v/v)	Solid-phase extraction with water and MeOH	0.25 ng/mL	10 ng/mL for plasma; 5 ng/mL for urine
Knych et al., 2014	Plasma	LC-MS/MS	C18 (2.1 mm × 500 mm, 3 μm)	ACN in H2O with 0.2% formic acid	Protein precipitation (methyl tert‑butyl ether)	0.05 ng/mL	0.1 ng/mL
Hovanessian et al., 2014	Plasma	HPLC-Fluorescence	C18 (3 mm × 150 mm, 3.5 μm)	ACN and 0.25% trifluoroacetate anhydride in H2O (45:55, v/v)	Protein precipitation, ACN	2.5 ng/mL	5 ng/mL
Holland et al., 2015	Plasma	HPLC-Fluorescence	C18 (4.6 mm x 150 mm, 5 µm)	ACN and 0.25% trifluoroacetate anhydride in H2O (50:50, v/v)	Solid-phase extraction with double deionized H2O and MeOH	2.5 ng/mL	5 ng/mL

NA: Not Available, LOD: Limit Of Detection, LOQ: Limit Of Quantification, ACN: Acetonitrile.

The clean up methods, as well the determined Limits of Detection (LOD) and Limits of Quantification (LOQ), are summarized in Table 2 as well.

## Pharmacology

### Pharmacokinetics

FX was developed specifically for horses by Merial Ltd.® as an oral paste formulation (branded as Equioxx®), with an intended therapeutic dose in horses of 0.1 mg/kg per day for up to 14 consecutive days (EMA, 2004). A formulation for intravenous injection is also available, of 0.09 mg/kg once daily for up to 5 days. The 57 mg canine tablet (Previcox®) has been reported for administration in horses for cost savings, but this is an off-label usage of the FX preparation (Holland et al., 2015). Extra-label drug usage in companion animals is permissible only when an approved medication has been determined by the attending veterinarian to be clinically ineffective for the labeled use, in which case another animal-approved drug may be used legally off-label. Economic considerations, however, are not viable reasons for using off-label drugs, unless a veterinarian chooses an approved human medicine to treat pain and suffering in non-food producing animals, even if an approved animal drug is available (Holland et al., 2015).

Merial Ltd.® has established the majority of the currently available safety information (NADA 141–253; FDA, 2005). Other evidences presented in horses are primarily related to PK factors and clinical trials for the use of FX in adult horses with OA. The main PK parameters of FX found in the literature concerning horses, are shown in Tables 3 and 4. FX, like other NSAIDs used to treat horses including phenylbutazone, flunixin meglumine, ketoprofen, and aspirin, is well absorbed, highly protein bound, and undergoes hepatic metabolism and renal excretion (Goodrich & Nixon, 2006; Kahn, 2005). However, FX, unlike these other NSAIDs, is a highly selective and potent COX-2 inhibitor with a much longer half-life, allowing for a once-daily dosing, which encourages compliance and provides longer-lasting pain relief.

**Table 3:** Summary of firocoxib experimental protocols in horses and safety studies published in the literature.

Reference	*n*	Species	Health status	Feed status	RoA and formulation	Dosage schedule	Safety data
Cox et al., 2012	6	Mixed-breed adult mares	Healthy	fasted for 12 h prior to treatment	Oral paste (in combination with 5 mg/kg enrofloxacin IV)	Single dose	No side effects noted
Cox et al., 2013	6	Mixed-breed adult mares	Healthy	Fed	Oral paste (0.82% (wt/wt) paste)	Multiple dose regimen; once daily for 10 days	No side effects noted
Wilson et al., 2017	6	Neonatal American Quarter Horse foals of mixed gender	Healthy	Fed (suckling)	A 20 mg/mL solution IV (Equioxx®, Merial)	Once daily for 7 days	No side effects noted
Letendre et al., 2008	6	Female horses (5 Paint horses and 1 Quarter Horse)	Healthy	Fed	Oral paste (0.82% (wt/wt) paste)	Once daily for 12 consecutive days	No side effects noted
	12	12 healthy male and female horses (predominantly Quarter Horse breed)	Healthy	Fed	IV administration of firocoxib (0.2 mg/kg) as a 2% (wt/vol) solution.	Once daily for 9 consecutive days	No side effects noted
Kvaternick et al., 2007	24 (12 PO, and 11 IV)	Males (some castrated) and females, unspecified breeds	Healthy	fasted for 12 h prior to treatment	The first 3 groups were given the oral paste, and the 4th group IV	Single dose	No side effects noted
Knych et al., 2014	9	3 Thoroughbreds, 4 Quarter Horses, one Westphalian and one Dutch Warmblood (5 geldings and 4 mares	Healthy	Fed	3-way crossover design of three groups, with the IV formulation, oral paste, and tablets	Multiple dose regimen (IV 5 for days; paste for 14 days; tablets for 14 days)	No side effects noted
Hovanessian et al., 2014	7	Neonatal American Quarter Horse foals of mixed gender (six colts and one filly; 36 h of age)	Healthy	Fed (suckling)	Oral paste (0.82% (wt/wt) paste)	Once daily for 9 consecutive days	No side effects noted
Holland et al., 2015	6	Adults, unspecified breeds	Healthy	Fed	3-way crossover design of three groups, with the IV formulation, oral paste (57 mg), and tablets (57 mg)	Single dose	No side effects noted
Kivett et al., 2014	6	Adults (15–20 years), unspecified breeds	Healthy	Fed	Oral paste (in combination with 2.2 mg/kg oral phenylbutazone)	Once daily for 10 consecutive days	No visual side effects noted (significant elevation in serum creatinine that may cause renal disease)

IV, intravenous; n, animal sample size; RoA, Route of Administration.

**Table 4:** Main pharmacokinetic parameters of firocoxib found in the literature in horses.

	Dose FX (mg/kg)	C_max_ (µg/mL)	T_max_ (h)	t_1/2_ kel (h)	Cl (mL/h/kg)	AUC_0-∞_ μg*h/mL	V_d_ (L/kg)	MRT (h)
Cox et al., 2012	0.1 mg/kg PO (alone)	0.091	0.87	31.53	/	2.37	/	41.21
	0.1 mg/kg PO (with 5 mg/kg enrofloxacin IV)	0.079	1.17	35.51	/	2.66	/	48.67
Cox et al., 2013	Loading dose of 0.3 mg/kg followed by nine daily doses of 0.1 mg/kg PO	0.183	9.24 (days, after the first administration)	41.76	/	12.5	/	ND
Wilson et al., 2017 (foals)	0.09 mg/kg IV (the shown data correspond to the 7th day of administration)	90.4 (C0)	/	15.9	96	1.96	1.79	22.2
Letendre et al., 2008	0.01 mg/kg PO (the shown data correspond to the final or 6th day of administration)	0.173	0.79	36.5	/	3.12	/	ND
	0.2 mg/kg IV (the shown data correspond to the 12th day of administration)	0.523 (C0)	/	44.2	40.5	5.5	2.3	ND
Kvaternick et al., 2007	0.1 mg/kg PO (F%: 79)	0.075	3.9	29.6	/	2.32	/	ND
	0.1 mg/kg IV	0.21 (C0)	/	33.8	36.7	2.98	1.69	ND
Knych et al., 2014	0.09 IV (data shown for the 5th day)	/	/	39.36	43.5	2.27	3.66	ND
	0.1 PO paste (data shown for the 14th day)	0.118	13.2 (days, after the first administration)	40.8	/	2.45	/	ND
	0.1 PO tablet (data shown for the 14th day)	0.137	13.3 (days, after the first administration)	41.52	/	2.68	/	ND
Hovanessian et al., 2014 (foals)	0.1 paste (data shown for day 9)	0.071	1.46	11.04	/	1.25	/	16.22
Holland et al., 2015	0.1–0.13 IV	/	/	31.07	42.61	2.55	1.81	45.22
	0.1–0.13 paste (57 mg) (F%: 111)	0.074	1.17	30.12	/	2.66	/	43.79
	0.1–0.13 tablet (57 mg) (F%: 87.79)	0.057	3.2	32.77	/	2.02	/	48.16

C_max_, peak plasma concentration; T_max_, time of peak concentration; t_1/2_ kel, terminal half‐life; Cl, plasma clearance; V_d_, volume of distribution; MRT, mean residence time; AUC_0-∞_, area under the concentration-time curve from dosing (time 0) till the time extrapolated at infinity; /, not applicable.

#### Bioavailability, absorption, plasma steady-state concentrations and accumulation

FX is rapidly and effectively absorbed in horses upon oral administration, which is the case for most NSAIDS since they are weak acids with high lipophilicity at physiologic tissue pH (Toutain & Bousquet-Mélou, 2004a). The first pass metabolism seemed low instead, based on the previously reported high bioavailability (F%) values (at a dose of 0.1 mg/kg) that ranged between 79% (Kvaternick et al., 2007) and 87% (Holland et al., 2015) for the canine tablets, and 111% for the equine paste (Holland et al., 2015), as reported in Table 4.

For single oral doses (or after the first given dose), the C_max_ values ranged between 0.045 (Letendre et al., 2008) to 0.183 µg/mL (Cox et al., 2013) at T_max_ values between 0.79 h (Hovanessian et al., 2014) and 7.8 h (Letendre et al., 2008). Horses that were fasted had a greater C_max_ and a shorter T_max_, as well as a higher AUC, when compared to horses that were not fasted (Kvaternick et al., 2007; Holland et al., 2015; Letendre et al., 2008; Knych et al., 2014). The presence of food in the oral cavity or stomach may reduce absorption. This could be due to direct physical obstruction of absorption or binding of the medication to cellulose in the feed, resulting in lower systemic plasma concentrations (Holland et al., 2015). Such differences of the PK parameters with changes in feeding conditions for horses have been reported for NSAIDs (Toutain & Bousquet-Mélou, 2004a; Jaussaud et al., 1992). Young et al. (2020) also assumed that adding the medications in grain may have contributed to the lack of efficacy of both gabapentin and FX, and that fasting before administration could be the optimum approach. Variations in bioavailability, and the extent of absorption, are also likely to be attributable to differences in the experimental design, and the difference between the given formulations. In Holland et al. (2015), the canine tablet bioavailability was lower than the equine paste (87.78 ± 54.32% *vs*. 111.71 ± 54.35%, respectively). As for the tablets, they were administered in 20 mL of water, plus 40 mL of corn syrup in Holland et al. (2015), compared to dissolution of the tablets in 4–5 mL of water only in the study by Knych et al. (2014). The difference in results could also be due to sensitivity of the chosen sample analysis method (the type of the used detectors), as well as the chosen time points for each study, since in some studies such as in (Holland et al., 2015, Letendre et al., 2008), the plasma concentrations were still well above the LOQ at the last time point, and thus a large percent of the AUC_0-∞_ had to be extrapolated, compared to other studies.

It should be noted that the PK parameters of the oral formulations varied between horses which are in the same study too. This is not surprising given that orally administered drugs typically have higher pharmacokinetic variability (Bouckaert et al., 1994; Soma et al., 2001) due to a variety of factors that may influence the absorption process, such as gastric emptying time, the presence of feed in the horse's mouth, buccal/sublingual absorption, formulation, and so on.

As for the multiple-dose regimen, a more rapid and more complete absorption was noted for the last administered doses, with significantly higher C_max_ and shorter T_max_ values than for the first given dose. Aside from the feeding pattern, which may have altered absorption, and the possibility of saturated first pass metabolism, it was explained by a high accumulation index after the last dose (Hovanessian et al., 2014; Letendre et al., 2008; Knych et al., 2014), that varied (oral paste) between 3.8 in Letendre et al. (2008) and 6.37 in Knych et al. (2014). Due to the long half-life (t_1/2_ kel) of FX, which is the primary determinant of the time to reach steady state plasma concentrations, and its slow elimination, it bio-accumulates with daily multiple doses and therefore concentrations at steady-state are 3–4 times higher with much less fluctuations than after a single dose (Kvaternick et al., 2007; Knych et al., 2014). The time to reach steady state would also be prolonged. In fact, following oral administration, steady state in adult horses was achieved by the 10^th^ consecutive dose for both formulations in Knych et al. (2014), and by the seventh daily dose in Letendre et al. (2008). In foals, in which t_1/2_ kel is much shorter than in adults (to be discussed later on), steady state was achieved after 4 doses (Hovanessian et al., 2014). Cox et al. (2013) demonstrated that near steady-state concentrations can be achieved with a single loading dose of 0.3 mg/kg FX oral paste at three times the label dose (0.1 mg/kg), compared to 7–10 days administration at the label dose (Letendre et al., 2008; Knych et al., 2014). This allowed the achievement of targeted average drug concentrations (with less fluctuations) at a steady state faster than a multi-dose regimen without a loading dose.

#### Plasma protein binding and distribution

Plasma protein binding is generally high for NSAIDs. The average binding of FX to horse plasma proteins at concentrations ranging from 0.25 to 1.0 µg/mL was 97%, and the variability in binding between concentrations was negligible, and thus only a small fraction is biologically active in the blood (Kvaternick et al., 2007). FX is broadly distributed in tissues, owing to its physicochemical features, which includes high lipophilicity and low ionizability at physiological pH. The distribution volume is substantially higher than for most NSAIDs (Letendre et al., 2008), with values ranging between 1.69 and 3.66 L/kg after IV administration (Table 4). The high lipophilicity of such drugs, as well as their high logP (logarithm of the lipid/water partition coefficient), favor diffusion across biological membranes for the unbound fraction of FX and therefore also intracellular distribution. This may also explain the long tissue half-lives, which can act as a reservoir (Grimm et al., 2015). The whole radio-residue study revealed that it is widely distributed throughout the body, even penetrating into the synovial fluid (approximately 30% of plasma concentration), and that is where its benefit in treating OA lies. A 14-day residue analysis (V. Kvaternick, unpublished data) verified its diffusion throughout the body, after that it was found in the liver, fat, kidney, and muscle. Tissue residues depleted in an approximately linear manner and at 14 days after the last dose were <5 ng/g in kidney and muscle tissues and 63.7 and 20.5 ng/g in liver and fat, respectively.

In horses following paracentesis, FX was shown by Hilton et al. (2011) to widely penetrate in healthy eyes and to a greater extent than flunixin meglumine. FX's lipophilic and non-ionizable properties account for its enhanced blood-aqueous barrier (BAB) and aqueous humor penetration. Even though the penetration of FX into the intraocular space during naturally occurring intraocular inflammation was not studied, FX, like most NSAIDs, penetrates rapidly into inflammatory tissue along with plasma proteins. Furthermore, because inflammation is associated with a breakdown in the integrity of the BAB, it is expected that the increased penetration of FX will be sustained and even augmented during naturally occurring disease.

#### Metabolism, metabolites, and excretion

Hepatic biotransformation is the primary mechanism of metabolism of FX, limited however. The metabolic pathways are *via* dealkylation (major metabolic pathway) and hydroxylation (minor pathway). FX is also glucurono-conjugated in the liver. After the hepatic metabolism, the excretion of the parent drug is mainly renal (Goodrich & Nixon, 2006) since approximately 68% of FX was recovered in the urine, and approximately 15% in the feces (Kvaternick et al., 2007).

In Kvaternick et al. (2007), in which 10 horses received 7 consecutive daily doses of radio-labelled FX compound at a dose of 0.3 mg/kg, the majority of radioactivity was excreted within 3 days after the last dose. The main metabolites are descyclopropylmethylfirocoxib (DFX) and the glucuronide conjugates, found mainly in kidneys alongside the dealkylated parent. A third metabolite discovered in urine was also identified as the hydroxylated parent's glucuronide conjugate. The glucuronide conjugate was likewise the predominant metabolite identified in urine and feces. DFX, on the other hand, was a large component of urine but not feces. FX was the major residue in all edible tissues (liver, muscle, fat and kidneys) and in feces. The ileum, bile, and jejunum contained the highest level of total radio-residues among the various tissues/fluids tested 3 days after the last dose, followed by the duodenum, large intestine, pancreas, cecum, bile duct, lung, and stomach. The radioactivity concentration in bile following 72 h after the final dose was almost 74% lower than at 6 h after the final dose, indicating that the FX and its metabolites were rapidly eliminated (Kvaternick et al., 2007).

These metabolites had low or no pharmacologic activity, as their metabolic pathways were assessed *in vitro*, and FX was incubated with rat liver microsomes, horse liver microsomes, and horse liver S9 fractions under oxidative conditions (Kvaternick et al., 2007). In fact, results showed DFX to be a much less potent COX inhibitor than FX *in vitro*. Concentrations >100 µM were required for inhibition (IC_50_) with DFX in the COX-2 human whole blood assay, compared with FX concentrations of 0.09 µM obtained from a horse whole blood assay (McCann et al., 2004). Except for prodrugs such as sulindac and nabumetone, which undergo reductive or oxidative transformations to become active derivatives, or phenylbutazone, which undergoes oxidative transformation to active hydroxyl‑metabolites such as oxyphenbutazone, the majority of NSAID metabolites, particularly glucuronide conjugates, are inactive or ineffective COX inhibitors (Mehanna, 2003). Glucuronidation produces more polar molecules, easily eliminated by the body, however lack the lipophilic characteristics needed to interact with the COX enzyme and prevent arachidonic acid binding. Furthermore, the bulky glucuronide molecule may be stereo-chemically prohibitive of binding in the active sites of the COX-2 or COX-1 isozymes. A study by Nicoll-Griffith et al. (2000), indicating the lack of activity of a glucuronide compound of a class-related medication, notably rofecoxib, lends weight to this notion.

#### Clearance, half-life and dose linearity

Total body clearance of FX in horses is relatively low and similar in magnitude to that for other NSAIDs, such as meloxicam, flunixin meglumine, phenylbutazone, and tolfenamic acid (Letendre et al., 2008; Toutain & Bousquet-Mélou, 2004b). Mean clearance values were similar between the various studies and ranged between 36.7 and 40.5 mL/h/kg for adult horses, as reported in Table 4. FX has accordingly a low extraction ratio (< 0.05) (Letendre et al., 2008), similarly to many other NSAIDs (Fadel et al., 2022). The parent drug's limited renal clearance is consistent with its low urine-to-plasma ratio of 0.35, where the FX concentration in urine samples decreased in parallel to the FX concentrations in the plasma samples. Maximum concentrations in urine were detected during the first 1–3 h after the last dose, and thereafter decreased exponentially and in parallel to concentrations in plasma, with a t_1/2_ kel of 35.3 ± 7.8 h after the final dose (Letendre et al., 2008).

FX's prolonged t_1/2_ kel is one of its most noteworthy PK properties (Kvaternick et al., 2007). It is approximately 5–30 times longer than that observed for other commonly used NSAIDs in horses, such as phenylbutazone, ketoprofen, and flunixin meglumine (Lees, 2009). This extended half-life is the result of the combination of the two previously discussed PK factors of FX: a large volume of distribution and a relatively low total body clearance. Because of this relatively long t_1/2_ kel in adults, ranging between 30.12 and 44.2 h (Table 4), and FX's high potency, once-daily administration is sufficient for prolonged treatment of animals with chronic pain and inflammation (Letendre et al., 2008; Hovanessian et al., 2014). FX also demonstrated linear kinetics after both single and multiple doses, as AUC and C_max_ ratios were dose proportional at 0.5–2 times the recommended dose (Kvaternick et al., 2007; Letendre et al., 2008). When the pharmacokinetic behavior of the drug is linear after multiple doses and the half-life is known, plasma concentrations at any time during the dosing regimen as well as the accumulation can be predicted accurately (Letendre et al., 2008).

#### Pharmacokinetic differences between neonates and adult horses

Similar to previous studies in which traditional NSAIDs were administered to equine neonates (Semrad et al., 1993; Wilcke et al., 1998; Crisman et al., 1996), FX displayed significant differences in its pharmacokinetics between adult horses and foals (Wilson et al., 2017; Hovanessian et al., 2014).

The key differences are the faster clearance and shorter t_1/2_ kel in foals, as was also the case with meloxicam in foals, in Raidal et al. (2013). There could be several explanations for the faster clearance (96 mL/h/kg, Wilson et al., 2017). Potentially, the effect of ontogeny and maturation of drug-metabolizing pathways in the liver of neonatal animals could account for a portion of the faster clearance. Raidal et al. (2013) hypothesized that meloxicam clearance was increased in neonatal foals because the cytochrome P-450 enzyme systems are among the most abundant liver enzymes, and thus younger animals have a relative abundance of these enzymes when liver volume is normalized to body weight, which increases drug clearance (Blanco et al., 2000; Burgos- Vargas et al., 2004). Additionally, the gastrointestinal tract, blood cells and potentially the lungs may be affecting the rate of FX metabolism in this age group (Allegaert et al., 2007). Furthermore, FX is a highly protein bound drug, and therefore, one mechanism of increased clearance in foals may be that lower plasma protein concentrations lead to a higher proportion of free drug, resulting in more free drug available for renal excretion (Toutain & Bousquet-Mélou, 2004b). This mechanism has led to increased clearance of drugs in human neonates, compared to adults (Yanni et al., 2011). Also, faster renal excretion of FX due to an increased glomerular filtration rate in foals compared with adult horses, and a different urinary pH (Gonda et al., 2003; Savage, 2008), may have accounted for an increased clearance.

The shorter t_1/2_ kel with an average of 13 h in foals, contrasted with 31 h in adults, may be related to either an increased clearance or a decreased volume of distribution (V_d_). However, foals typically have a higher V_d_ compared with adults due to a higher percent body composition of water, and this would tend to increase the half- life (Hovanessian et al., 2014). Furthermore, as shown in Table 4, there is no significant difference in the V_d_ between foals and mature horses. As a result, the lower t_1/2_ kel can be mainly attributed to the faster clearance in foals.

After single doses, T_max_ was considerably shorter in foals compared with adults (Hovanessian et al., 2014). This feature of faster absorption may be explained by dietary differences between adults and foals. FX may adhere to milk proteins in the foal's digestive tract, facilitating its passage into the bloodstream (Hovanessian et al., 2014). Consequently, C_max_ was also higher as more of the drug is absorbed in a shorter period. As for the multiple-dose regimen, foals did not show an elevated C_max_ after achieving the steady-state plasma levels, as adult horses did. This is due to the shorter t_1/2_ kel and the faster elimination of FX, resulting in significantly reduced accumulation in foals with a mean estimated accumulation index of 1.29 (Hovanessian et al., 2014).

### Pharmacodynamics and therapeutic effects

Like other coxibs, FX's COX-1 active site is smaller than that of COX-2, because of its steric hindrance. The relatively narrower channel of the COX-1 active site and the non-linear configuration of the COX-2 selective drug do not allow significant binding of the COX-2 drugs such as FX to the COX-1 active sites (Kim & Giorgi, 2013; Fogle et al., 2021). The drug selectivity for COX-2 is determined through calculation of the inhibitory concentration (IC_50_) COX-1:COX-2 ratio (Bergh & Budsberg, 2005). Accordingly, FX is a very selective COX-2 inhibitor (Stichtenoth & Frolich, 2003), with COX-1/COX-2 IC_50_ ratios of 643 for horses, 384 for dogs, and 58 for cats, as shown with *in vitro* models, values that are one to two orders of magnitude higher than those reported for other coxibs or nonselective NSAIDs, and thus pointing out a favorable safety margin (Brideau et al., 2001, Lees et al., 2004, McCann et al., 2004, Ricketts et al., 1998). In fact, FX concentrations that yield 80%, 95%, and 100% inhibition of COX-2 activity produce 0%, 0%, and 3% inhibition of COX-1 activity, respectively (McCann et al., 2004). This COX-2 inhibition is denoted by a decrease in prostaglandin (PGE2) concentrations, consistent in all the previous studies. In fact, no significant differences in PGE2 levels between the four drugs—FX, meloxicam, phenylbutazone, and flunixine meglumine—were found by Fogle et al. (2021), as it is similar in other studies (Doucet et al., 2008; Cook et al., 2009). Similar COX-2 inhibition levels for all four medications would indicate similar analgesic and anti-inflammatory effects, which is contrary to some veterinarians’ clinical impression of inferior analgesia with FX (and meloxicam) compared to conventional non-selective NSAIDs.

According to *in vitro* whole blood tests, the concentration of FX needed to reach IC_50_ in equine whole blood that has been incubated with LPS is around 30 ng/mL, with an associated IC_80_ of 67 ng/mL (McCann et al., 2004; Kvaternick et al., 2007). The IC_80_ is equal to 103 ng/mL when data from the whole blood *in vitro* assay is corrected for plasma concentrations, assuming that the plasma concentration is 65% of the whole blood (Barton et al., 2014). Accordingly, in Holland et al. (2015), the plasma concentrations achieved, following IV administration of a single dose of 0.1 mg/kg, were similar to this previously mentioned IC_80_. It was also similar to that achieved following oral administration of multiple doses of either the paste or tablet formulation (0.1 mg/kg; Barton et al., 2014). In all the routes of administration, however, reaching IC_80_ did not occur until 24 h after drug administration. This response might indicate that this drug is not as effective as some other NSAIDs in cases of acute inflammation (Holland et al., 2015). Moreover, this emphasizes the need for a loading dose to shorten the time to achieve a therapeutic response, as mentioned previously (Holland et al., 2015).

In the single dosage regimen in Holland et al. (2015), over a 48-hour period, the IV formulation significantly outperformed the oral tablet formulation in terms of COX-2 inhibition. Additionally, the paste had a far greater effect than the oral tablet. But in the prior investigation by Barton et al. (2014), employing a comparable *ex vivo* model, equivalent PGE2 concentrations were observed between oral tablet and paste FX-treated groups. This is because in the last mentioned study, they administered seven consecutive daily doses of the drug prior to determining the COX-2 inhibitory effect, and with the previously discussed accumulation factor, any differences in COX-2 inhibition between oral formulations are likely negated by the higher concentrations achieved after multiple doses (Holland et al., 2015).

As for the thromboxane (TBX2), no appreciable inhibition of its concentration was noted (Holland et al., 2015), confirming its COX-1 sparing effect. In fact, at the 8 and 12 h time points after administration of all three FX formulations, TXB2 concentrations rose. This increase in TXB2 was also noted at 12 h in Fogle et al. (2021), and it may be caused by a COX-1 response, maybe through a negative feedback loop, to a relative lack of prostanoids coming from COX-2. TXB2 levels were considerably greater in the 48 h following the third dosage of FX and meloxicam than in horses given flunixin meglumine or phenylbutazone (Fogle et al., 2021). Treatment with FX allowed an increase in plasma TXB2 concentrations after surgery in Cook et al. (2009) as well, whereas this increase was inhibited by treatment with flunixin meglumine. This rise in COX-1 in ischemic-injured mucosa at 18 h was partially reversed by FX therapy, a finding that has been reported for meloxicam as well (Little et al., 2007), a preferential COX-2 NSAID.

Concerning the half maximal effective concentration (EC_50_), it represents the affinity of a drug for binding to the receptor (Toutain & Cester, 2004). It was reported to be 0.027 ug/mL for FX (Holland et al., 2015), which has the highest affinity for its receptor compared to meloxicam (0.13–0.195 ug/mL, Toutain & Cester, 2004), flunixin meglumine (0.2–0.9 ug/mL, Toutain et al., 1994), and phenylbutazone (1.5–4.3 ug/mL, Toutain et al., 1994). FX remained above the EC_50_ up to 48 h (Fogler et al., 2021).

As for the therapeutic effect, FX is approved for use in horses to control pain and inflammation associated with OA. In Back et al. (2009), the American Association of Equine Practitioners (AAEP) 0–5 lameness scale and force-plate measurement were used to assess the lameness of 64 horses, of which 33 were assumed to have OA and 31 to have navicular syndrome, before oral paste FX administration on days 0 (first day of administration), 2, and 6. The horses who received 0.5 mg/kg on day 6, 0.01 mg/kg on days 2 and 6, and 0.25 mg/kg on days 0 through 6 showed a substantial increase in peak vertical force (a sign that the horses were less lame). The authors of this report consequently concluded that force-plate analysis objectively identified that 0.1 mg/kg was an effective dose of FX to decrease chronic lameness.

In Orsini et al. (2012), 390 of 429 horses with OA-related musculoskeletal pain and lameness from 80 different sites were examined for improvement in lameness, after administering the oral paste for 14 days at a dose of 0.1 mg/kg. Clinicians reported improvement in 79% of horses at the end of the study with the most rapid improvement being within 7 days after starting the treatment.

In other efficacy studies as well, FX has been compared to many NSAIDs for the treatment of lameness in horses. A randomized controlled clinical trial with 253 horses with naturally occurring OA, including navicular disease, compared the efficacy and safety of paste formulations of FX and phenylbutazone (Doucet et al., 2008). All horses were treated with either FX (0.1 mg/kg PO, q 24 h) or phenylbutazone (4.4 mg/kg PO, q 24 h) for 14 days. At the conclusion of the research, 85% of the FX-treated horses had improved, with no significant difference in the clinical lameness scores improvement compared to phenylbutazone (87%). However, FX was shown to be potentially having a greater clinical improvement than phenylbutazone for focal pain, joint effusion, and range of motion, at least when administered for 14 days.

In another field trial (Koene et al., 2010), 96 horses with a lameness grade of at least 2 (AAEP lameness scale), were divided into two equal treatment groups, with one group receiving oral paste FX (0.1 mg/kg q 24 h), or a single loading dose (2 mg/kg) then twice-daily dose (1 mg/kg) of oral paste vedaprofen, each for 14 days. At 14 days, 83% of horses treated with FX showed clinical improvement, compared to 65% of horses treated with vedaprofen. According to the authors, the oral paste version of FX was found to be extremely efficacious, well-tolerated, and acceptable for the treatment of pain and inflammation associated with lameness in horses, as was the case in the other studies.

In a prospective blinded trial employing the reversible lameness model, Foreman et al. (2015) found that a three-fold loading dose of FX (0.27 mg/kg IV once) was comparable to phenylbutazone (4.4 mg/kg IV twice a day) in terms of effects on heart rates and lameness scores throughout a 24-hour monitoring period. However, when the trial was done without a loading dose, FX was no better than saline placebo in effects on heart rate or lameness score, in 24 h. Moreover, these studies, alongside the *in vitro* studies (IC_80_; Holland et al., 2015), emphasize the need for a loading dose to shorten the time to achieve a therapeutic response, especially in acute illnesses, as mentioned previously (Holland et al., 2015).

Aside from OA and lameness, administration of FX resulted in effective visceral analgesia while permitting recovery of mucosal barrier function in horses undergoing ventral midline celiotomy and jejunal ischemia-reperfusion injury without enterectomy, in Cook et al. (2009). FX was also equally effective as flunixin meglumine in reducing PGE2 synthesis caused by ischemia-reperfusion damage in that study. Also, in horses recovering from small intestine strangulation surgery, FX (0.3 mg/kg loading dose, followed by 0.1 mg/kg labelled dose every 24 h) provided analgesia comparable to flunixin while protecting COX-1 homeostatic mechanisms required for renal and gastrointestinal repair (Ziegler et al., 2017).

FX did not reduce *ex vivo* COX-1 activity in horse patients undergoing elective surgery such as arthroscopy, according to Duz et al. (2015), and it could provide adequate analgesia and an alternative for the management of pain and inflammation in patients who cannot use non-selective NSAIDs. It could be also used to manage illnesses such as intestinal ischemia, right dorsal colitis, or pre-renal or intrinsic acute renal failure, colic and so on. Horses particularly suffering from systemic inflammatory response syndrome (SIRS) secondary to gastrointestinal disease, with pre-existing or concurrent renal compromise, may benefit from FX, rather than a conventional or COX-2 preferential NSAID (Fogle et al., 2021). However, more investigations are needed to approve its use in such circumstances.

## Safety evaluation

When taken alone at the prescribed dose (0.1 mg/kg for up to 42 days), there has been a very low rate of documented side effects for oral and IV administration of FX (Donnell & Frisbie, 2014; Doucet et al., 2008; Hovanessian et al., 2014; FDA, 2005). When given oral paste FX at the prescribed dose, four of 476 (0.9%) horses experienced lip edema, brief episodes of colic, mild oral ulcerations, lethargy, and sedation, and one of 48 (2%) horses experienced labial and tongue edema and hypersalivation (Koene et al., 2010). Six horses were given FX (0.1 mg/kg) and phenylbutazone (4.4 mg/kg) for ten days and their creatinine and total protein levels increased, indicating renal impairment, in Kivett et al. (2014). Besides this study, no side effects in horses were noted in the previously performed pharmacokinetic studies, as seen in Table 4.

Target animal-safety studies revealed oral ulceration in horses given FX oral paste at three and five times the recommended dose for 42 days, as well as clinical chemistry and coagulation disturbances in horses given five times the recommended dose for 42 days (FDA, 2005). In a subsequent research, all horses had delayed healing of preexisting oral ulcers, and one of eight horses developed renal papillary necrosis, when administered FX paste at the recommended dose. In horses given one to five times the approved dose of FX, IV administration has been linked to injection-site edema and perivascular inflammation (FDA, 2010).

To summarize, when used at the recommended doses, FX has relatively few side effects. With no reports of gastric ulceration as an adverse effect after prolonged administration, there is a widespread clinical view that FX is definitely a safe option to non-preferential NSAIDs, particularly in horses with gastrointestinal ulceration.

## FX use in racehorses, plasma regulatory threshold concentrations, and withdrawal time

NSAIDs use in performance horses is regulated due to their capacity to influence performance and potential to allow a horse to compete when it should not. The Racing Medication and Testing Consortium (RMTC) and the United States Equestrian Federation (USEF) are in charge of formulating threshold guidelines for race and performance horses in the United States, respectively. The goal is to set plasma or urine thresholds at which the drug has little or minimal pharmacological activity and can be effectively managed (Knych et al., 2014; EMEA, 2010; Toutain, 2010).

The RMTC proposes that the use of FX in plasma or serum be controlled in the United States, with a regulatory threshold concentration of 20 ng/mL for racehorses. This is based on a 7-day oral dosing at the FDA-approved label dose of 0.1 mg/kg. As for the European countries that follow the European Horserace Scientific Liaison Committee (EHSLC) recommendations, FX is controlled in urine with a suggested detection time of 15 days following dose of 0.1 mg/kg for 7 days.

The recommendation for non-racing performance horses is quite different. In the United States, the current plasma regulatory threshold concentration set by the USEF is substantially higher than that proposed by the RMTC (240 ng/mL). Conversely, in Europe, the International Equestrian Federation (FEI), recommends the same detection time in urine as the EHSLC (Toutain, 2010).

Afterwards, Knych et al. (2014) demonstrated that all three formulations, including the non-approved tablet form, when used at the recommended doses, fell below the USEF 2014 threshold (240 ng/mL) for the entire sampling period in all horses, and below the Racing Medication and Testing Consortium threshold (20 ng/mL) in all horses 7 days after administration of the final dose, and thus the recommended doses imply compliance to the guidelines provided by the relevant authorities.

Because FX can be used as a palliative for a variety of pathologies and inflammatory processes due to its demonstrated safety, it can result in indiscriminate use in sport horses. And since it is commonly administered in performance horses, it is critical to consider drug use regulation so that these concentrations do not exceed their limits, which could harm the animal not only in adverse effects of the drug, but also when affected sport horses are forced to compete, they may be exposed to developing more severe pathologies than those presented previously (Rangel-Nava et al., 2019).

To note finally, the withdrawal period is the allowed window of time between the last drug administration and the animal's slaughter, before any additional meat or milk is intended for human consumption. Concerning the meat withdrawal time, horses should only be butchered after 26 days following the final day of Previcox® medication (when treated with the oral paste). However, the use of Previcox® is not permitted in mares producing milk for human consumption (EMA, 2008).

### Drug-drug interactions

When FX first became available for use in horses in the United States, the USEF approved its usage in conjunction with other NSAIDs. Since then, this has been amended and currently FX cannot be combined with any other NSAIDs. Given the present understanding of COX selectivity, co-administration of FX with a nonselective NSAID is likely to impair the intended benefits of COX-2-selective NSAIDs and is hence not recommended (Ziegler et al., 2017). Additionally, the observed side effects from co-administrating NSAIDs imply that these combinations may increase the likelihood of complications, as it was the case in Kivett et al. (2014). Also, FX should not be combined with corticosteroids, diuretics, nephrotoxic medications, and highly protein-bound medications (USPC, 2007).

Given the common combination of enrofloxacin and FX in horses, it is important to note that the co-administration of oral FX and intravenous enrofloxacin did not affect the pharmacokinetic variables for either drug (Cox et al., 2012).

## Conclusion

*In vitro* and *in vivo* studies have demonstrated that FX is a highly selective COX-2 inhibitor. Both an injectable formulation for intravenous administration at a dose of 0.09 mg/kg for five days and an oral paste formulation with a dose of 0.1 mg/kg for 14 days are approved for use in treating OA in horses. A canine tablet formulation is also available, and may be an equally effective anti-inflammatory drug compared to the other formulations. However, the use of the canine formulation is controversial because it is unapproved, yet it is often provided because it is less expensive than the paste. FX is well absorbed in horses, with an oral bioavailability of 80% or more. It is slowly eliminated, and V_d_ values are around 2 L/kg. Because of the long elimination half-life, which has been reported to be as long as two days, it may take several days to achieve steady concentrations and maximal efficacy. To remedy this, a single 0.3 mg/kg loading dose has been recommended, which allows for the establishment of average steady-state drug concentrations within 24 h, making it suitable for acute treatment as well. It should be noted that FX in neonates displays different pharmacokinetics than in adult horses. After 14 days of dosing in clinical trials on horses with spontaneously occurring OA, the overall clinical efficacy of FX was optimal at the recommended doses.

## Data availability statement

The data sets used and/or analyzed during the current study are available from the corresponding author, upon request.

## Ethical statement

This manuscript is a review and does not need any ethical statement

## Declaration of Competing Interest

The authors declare that they have no known competing financial interests or personal relationships that could have appeared to influence the work reported in this paper.
